# Tailoring the sampling time of single-sample GFR measurement according to expected renal function: a multisite audit

**DOI:** 10.1186/s40658-022-00500-z

**Published:** 2022-10-26

**Authors:** Helena McMeekin, Sam Townrow, Mark Barnfield, Andy Bradley, Ben Fongenie, Daniel R. McGowan, Matthew Memmott, Charlotte A. Porter, Fred Wickham, Nick Vennart, Maria Burniston

**Affiliations:** 1 Barts Health NHS Trust, London, UK; 2 Leeds Teaching Hospitals NHS Trust, Leeds, UK; 3 Manchester University NHS FT, Manchester, UK; 4 Royal Free London NHS FT, London, UK; 5 Oxford University Hospitals NHS FT, Oxford, UK; 6 University of Oxford, Oxford, UK; 7 South Tyneside and Sunderland NHS FT, Gateshead, UK

**Keywords:** Glomerular filtration rate, GFR, Single sample, Multisite audit, Renal function

## Abstract

**Background:**

The 2018 BNMS Glomerular Filtration Rate (GFR) guidelines recommend a single-sample technique with the sampling time dictated by the expected renal function, but this is not known with any accuracy before the test. We aimed to assess whether the sampling regime suggested in the guidelines is optimal and determine the error in GFR result if the sample time is chosen incorrectly. We can then infer the degree of flexibility in the sampling regime.

**Methods:**

Data from 6328 patients referred for GFR assessment at 6 different hospitals for a variety of indications were reviewed. The difference between the single-sample (Fleming) GFR result at each sample time and the slope–intercept GFR result at each hospital was calculated. A second dataset of 777 studies from one hospital with nine samples collected from 5 min to 8 h post-injection was analysed to provide a reference GFR to which the single-sample results were compared.

**Results:**

Recommended single-sample times have been revised: for an expected GFR above 90 ml/min/1.73m^2^ a 2-h sample is recommended; between 50 and 90 ml/min/1.73m^2^ a 3-h sample is recommended; and between 30 and 50 ml/min/1.73m^2^ a 4-h sample is recommended. Root mean square error in single-sample GFR result compared with slope–intercept can be kept less than or equal to 3.30 ml/min/1.73m^2^ by following these recommendations.

**Conclusion:**

The results of this multisite study demonstrate a reassuringly wide range of sample times for an acceptably accurate single-sample GFR result. Modified recommended single-sample times have been proposed in line with the results, and a lookup table has been produced of rms errors across the full range of GFR results for the three sample times which can be used for error reporting of a mistimed sample.

## Introduction

The 2018 BNMS GFR guidelines [1] recommend a single-sample technique for routine measurement of GFR. This is a significant change compared with the previous BNMS GFR guidelines [2], which recommended a slope–intercept technique requiring between 2 and 4 samples. The multiple sample slope–intercept technique prevails in UK nuclear medicine departments; a national audit conducted in 2013 reported that 58 out of 59 responding centres employ a multiple sample technique with either two, three, or four samples [3]. Changing practice from multiple to single-sample GFR confers many benefits, both in terms of patient comfort and saved departmental resources but requires logistical changes which could limit compliance with the 2018 guidelines. One of these is the timing of the single blood sample. We aim to address this concern with this paper.

It is well established from theory that the accuracy of a single-sample GFR result depends on the time at which the sample was taken: the lower the GFR the later the sample should be taken for an accurate result [4]. This necessarily means that the expected renal function must be known before the test, a seemingly paradoxical situation. The 2018 BNMS guidelines recommend that the sampling time is set according to the estimated BSA-normalised GFR, giving five ranges with appropriate sampling times (Table 1 in the guidelines). They describe the use of serum creatinine measurements (eGFR), previous GFR measurements, and the patient’s history and clinical condition to inform the estimated GFR, and therefore the sampling time. However, we cannot depend on the accuracy of these estimates. In this work, we will investigate the range of single-sample times for an acceptably accurate GFR result and quantify the error contributed by a mistimed sample.

**Table 1:** Number of GFR results which were excluded from the analysis for QC or methodological reasons

Check	Sample timing or fewer than three samples	*R*^2^	Volume of distribution
Number of GFR studies removed	3038	263	628

## Methods

Data from 6328 patients referred for GFR assessment at six UK hospitals for a variety of indications were reviewed. The difference between the single-sample (Fleming) [5] GFR result at each sample time and the slope–intercept GFR result at each hospital was calculated. The slope–intercept calculation method recommended in the previous BNMS guidelines was applied to all GFR measurements, and the results were body surface area and Brochner–Mortensen corrected [2].

To ensure that only good-quality data were used in the analysis, data points were excluded if any of the following conditions were met: if any sample was taken outside a twenty-minute window around the intended time; if fewer than three blood samples were taken (minimum of three samples between 2 and 6 h post-injection); if the *r*^2^ correlation coefficient for the fit including all samples was less than 0.985; if the calculated volume of distribution was not in the range of 6–11.2 multiplied by the BSA. The 6328 initial studies were reduced to 2399 after these checks, please see Table 1 for details.

The volume of distribution check was based on the 2004 BNMS guidelines recommendation of 8 times the BSA with a 25% uncertainty margin; however, it was found that too many studies were excluded if this was applied, so a 40% upper margin was used. We are not worried that this has influenced the results by including studies with unreliable data: the large inherent variability in the relationship of BSA with volume of distribution is well documented; for example, Fleming and colleagues have recommended a 40% margin around the expected volume of distribution for GFR quality control checks [6].

The same analysis was performed on a dataset from one hospital which included a reference GFR calculated using a nine-sample area-under-the-curve (AUC) calculation, with samples from 5 min to 8 h post-injection (*n* = 418 studies after QC checks). The dataset and calculation method have been previously described [7]. The single-sample results were compared to the reference GFR rather than a slope–intercept GFR.

## Results

**Fig. 1:**
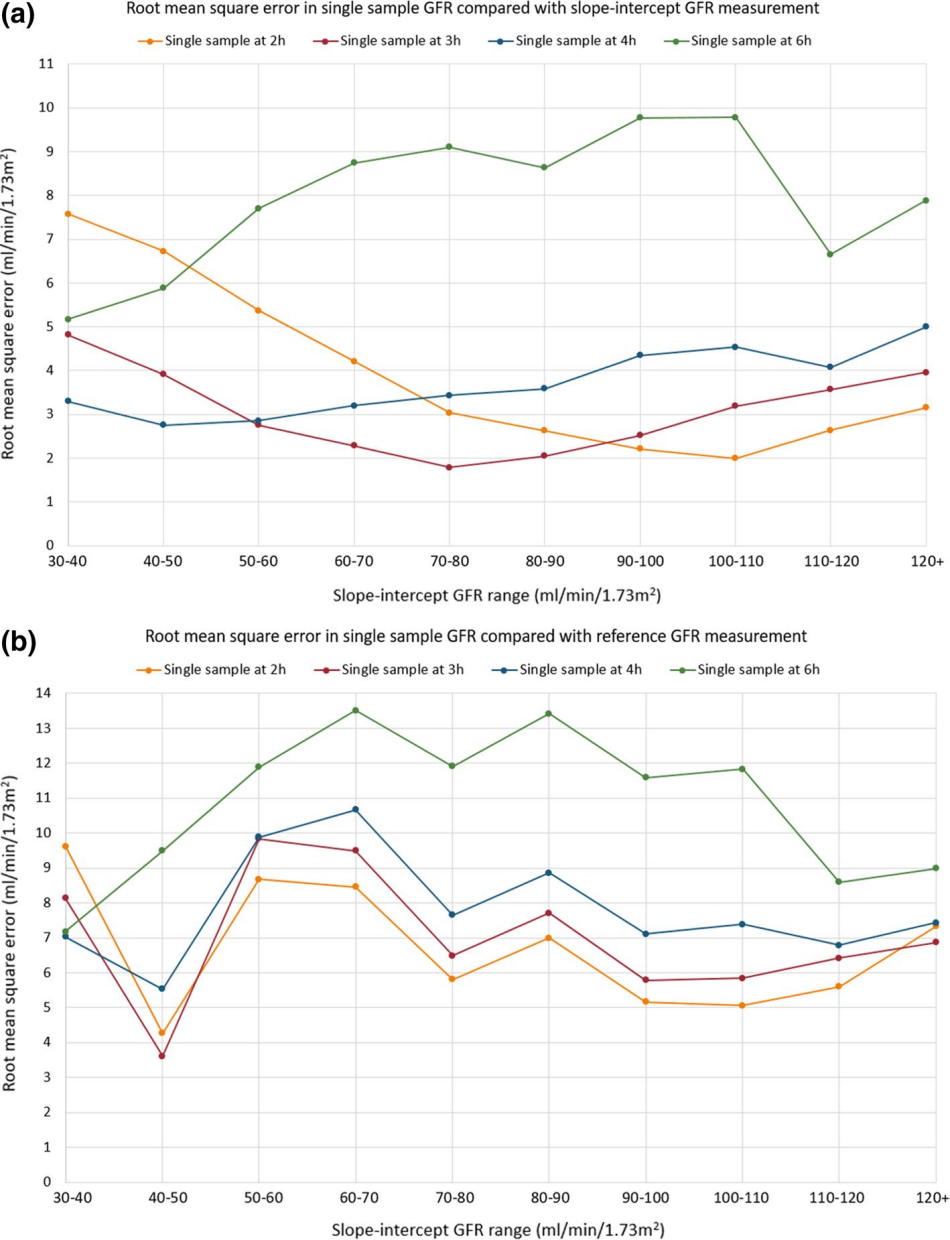
**a** Root mean square error in units of ml/min/1.73m^2^ in single-sample GFR calculated at four time points compared with slope–intercept GFR for each slope–intercept GFR range. **b** Root mean square error in units of ml/min/1.73m^2^ in single-sample GFR calculated at four time points compared with reference GFR for each reference GFR range. The y-axis scale has been extended compared with **a** to best display the data

**Fig. 2:**
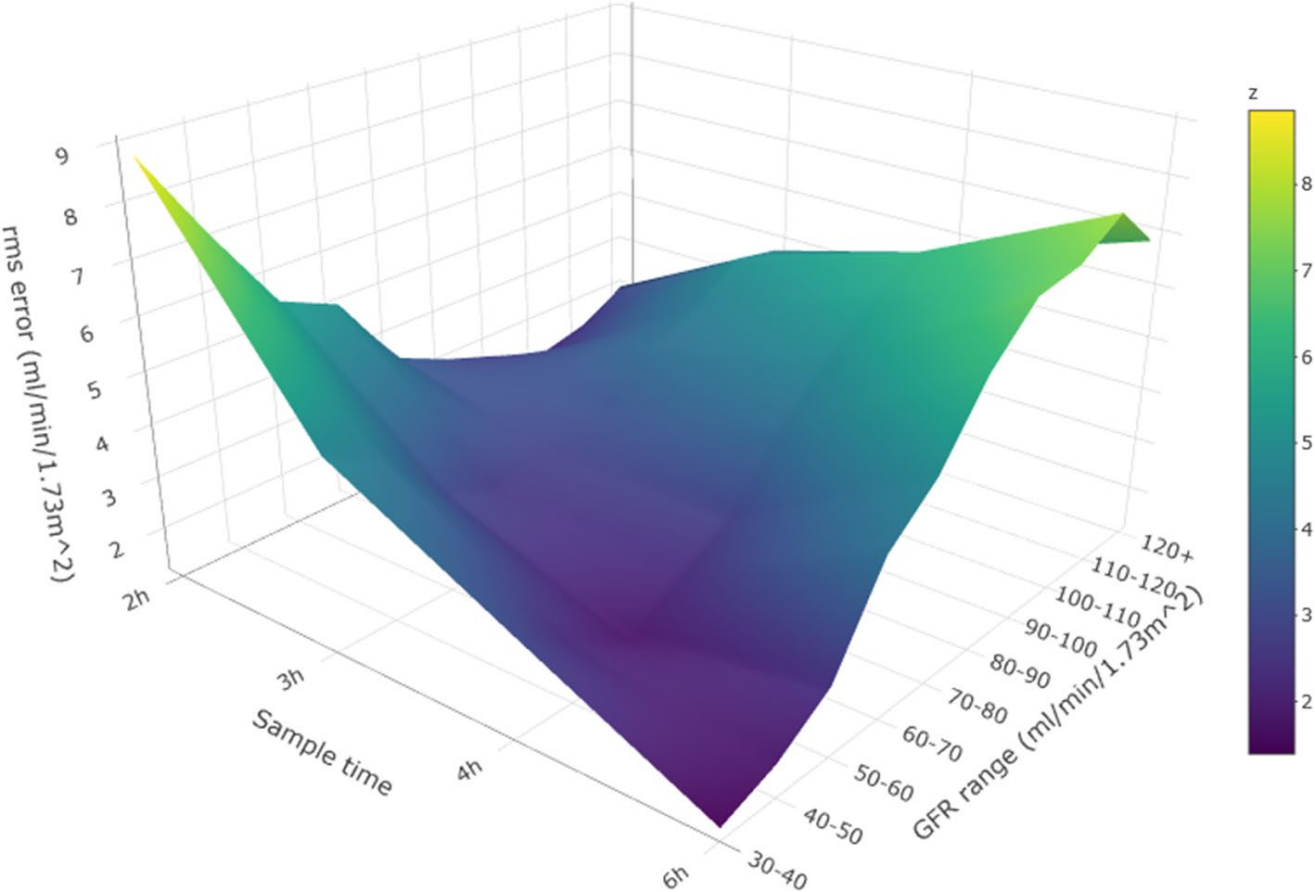
A 3D surface plot showing the rms error in units of ml/min/1.73m^2^ between single-sample GFR and slope–intercept GFR plotted against GFR range and single-sample time. The colours represent increasing rms error from purple to yellow on the colour scale

For the comparison between single-sample GFR and slope–intercept GFR, all GFR ranges contain more than 100 patient studies, apart from 110 to 120 and 120 + mL/min/1.73m^2^ which contain 97 and 88 patient studies, respectively. Table 2 provides the root mean square (rms) error in units of ml/min/1.73m^2^ between single-sample GFR and the slope–intercept GFR measurements. These data are represented graphically in Fig. 1a and as a 3D surface plot [8, 9] in Fig. 2. As predicted from theory, the time for the most accurate single-sample result increases as the GFR decreases. The rms error for a single-sample GFR result can be kept lower than or equal to 3.30 mL/min/1.73m^2^ by choosing an appropriate sample time. The 6 h single-sample time conferred benefit for none of the GFR ranges.

**Table 2:** Root mean square (rms) error in units of ml/min/1.73m^2^ between single-sample GFR and slope–intercept GFR measurements

Slope–intercept GFR mL/min/1.73m^2^	Sampling time (h)			
	2	3	4	6
30–40	7.57	4.82	**3.30**	5.17
40–50	6.73	3.91	**2.76**	5.89
50–60	5.37	**2.76**	2.86	7.70
60–70	4.21	**2.28**	3.20	8.74
70–80	3.04	**1.78**	3.43	9.10
80–90	2.63	**2.05**	3.59	8.64
90–100	**2.22**	2.52	4.35	9.78
100–110	**1.99**	3.19	4.54	9.78
110–120	**2.64**	3.57	4.07	6.65
120+	**3.16**	3.96	5.00	7.89

Lowest values are highlighted in bold text

Table 3 provides the rms error for the comparison of single-sample GFR and with the reference GFR and the number of patient studies. These data are represented graphically in Fig. 1b. For Fig. 1b, the y-axis scale has been extended compared with Fig. 1a to best display the data. Root mean square errors are larger across the board for the comparison of the single sample with the reference GFR. The most accurate sampling time is shifted lower compared with the slope–intercept GFR comparison.

**Table 3:** Root mean square (rms) error in units of ml/min/1.73m^2^ between single-sample GFR and reference GFR measurements

Reference GFR mL/min/1.73m^2^	Number of patient studies	Sampling time (h)			
		2	3	4	6
30–40	2	9.62	8.14	**7.02**	7.18
40–50	3	4.27	**3.62**	5.53	9.49
50–60	15	**8.67**	9.84	9.88	11.89
60–70	37	**8.45**	9.49	10.66	13.51
70–80	49	**5.81**	6.49	7.65	11.91
80–90	61	**7.00**	7.71	8.86	13.41
90–100	67	**5.17**	5.78	7.11	11.58
100–110	69	**5.07**	5.85	7.39	11.82
110–120	56	**5.61**	6.42	6.79	8.60
120+	59	7.33	**6.86**	7.43	8.99

Lowest values are highlighted in bold text

## Discussion

The data support the approach of the guidelines and demonstrate a reassuringly wide range of sample times for an acceptably accurate single-sample GFR result.

The results of this study suggest slightly different recommended single-sample times compared with the 2018 guidelines. Taking the lowest rms error sample time for each GFR range from Table 2, the comparison of single-sample and slope–intercept GFR produces the new recommendations in Table 4. Table 4 also includes the results from previous work [10] which recommends a 24 h sample time for GFR less than 25 mL/min/1.73m^2^. We have extended this to 30 mL/min/1.73m^2^ based on clinical experience to cover the full range.

**Table 4:** Proposed recommended single-sample times to use with the Fleming formula based on expected BSA-normalised GFR

Expected BSA-normalised measured GFR (mL/min/1.73m^2^)	Recommended single-sample time (h)
> 90	2
50–90	3
30–50	4
< 30	24

For the GFR range where 6 h sampling was recommended (30–50 mL/min/1.73m^2^), 4 h sampling has lower rms error compared with 6 h sampling, so the recommendation for 6 h sampling has been removed entirely. We recommend that the 6 h sample time is replaced with 4 h sampling in a revised version of the guidelines in the interests of accuracy and simplified departmental logistics. We hope that this simplified sampling regime will increase the routine use of single-sample GFR in hospitals. The sampling regime could be further simplified by omitting one or more of the recommended sampling times in accordance with the error level considered acceptable locally. Table 2 provides the necessary information on which this decision could be based.

One immediately obvious feature from Fig. 1b is how far away the single-sample, and by inference the slope–intercept GFR result, is from the reference GFR; rms errors are larger across the board for the comparison of single-sample with the reference GFR. We believe this to be due to the overestimation of GFR by the abbreviated techniques; the clearance of tracer from the plasma has not yet reached a terminal exponential at the start of sampling, so the gradient of a slope–intercept measurement flattens as 8 h is reached. This is compensated for at high GFR by the inherent underestimation due to the Brochner–Mortensen correction reaching a maximum value. It is not the purpose of this work to propose an improved single-sample formula to correct for this, and such a systematic difference in GFR results would have a far-reaching clinical impact which it is not practical to implement. Our primary focus is to provide an equivalent accuracy single-sample GFR to the slope–intercept method in current clinical use. The very low patient numbers in the 30–40 mL/min/1.73m^2^ and 40–50 mL/min/1.73m^2^ GFR ranges (2 and 3, respectively, see Table 3) make the analysis unreliable here, contributing to the odd appearance of the graph.

To predict GFR, we recommend using a recent eGFR (estimated GFR) result calculated from a serum creatinine blood test, if one exists. A 2019 study [11] found that an eGFR threshold of 40 mL/min/1.73m^2^ was appropriate for selecting patients where the GFR was subsequently measured as less than 25 mL/min/1.73m^2^. There is the possibility for confusion here, since we have recommended from this study a sample time of 4 h for an expected measured GFR 30–40 mL/min/1.73m^2^, counteracting the recommendation from the 2019 study. The large variability in eGFR is the culprit here. In an audit (unpublished) of our clinical practice, we found that a measured GFR (same day slope–intercept GFR with 2 h, 3 h, 4 h samples) of less than 35 mL/min/1.73m^2^ merited the patient returning for a 24 h sample on the next morning. In those cases, the 4-sample slope–intercept GFR (2 h, 3 h, 4 h, 24 h sample times) could give a result more than 20% lower than the same day result. Our recommendation in this tricky area would be for a 4 h single-sample measurement, with the possibility to bring the patient back for 24 h sampling depending on the same day result. To this end, we avoid scheduling these patient studies for Fridays.

Pertinent to this discussion is the inherent variability in GFR measurement: estimates of the repeatability of GFR measurement on the same patient over time suggest a variation of approximately 10% [12] and a study by Wilkinson et al. found a coefficient of variation of 12% in duplicate measurements of the same patient when permitted free exercise, and 8% when at rest [13]. The differences we have measured between GFR calculated with slope–intercept and single-sample techniques are within these patient dependent variations.

If no previous eGFR measurement is available, the clinical indication for the GFR test can guide the choice. A 2013 UK audit [3] of GFR measurement reported the following referral reasons: oncology patients for assessment pre-chemotherapy (70%); potential live renal donor (16%); monitoring of chronic kidney disease (10%); others (4%). Hopefully it is clear from the referral reason whether reduced renal function is suspected. If the referral is for monitoring of chronic kidney disease, then there is reason to expect reduced renal function, whereas if the clinical indication is first assessment pre-chemotherapy or live renal donor, then the choice of single-sample time can be guided by the normal range of GFR for the patient age.

In the event of a mistimed sample, that is when the GFR measured by the Fleming single-sample method is unexpectedly different from the pre-test predicted GFR, the rms errors given in Table 2 can be used as a lookup to report the error in comparison with multiple sample slope–intercept GFR.

## Conclusion

The results of this multisite study demonstrate a reassuringly wide range of sample times for an acceptably accurate single-sample GFR result. Modified recommended single-sample times have been proposed in line with the results which should aid clinical implementation. A lookup table has been produced which can be used for error reporting of a mistimed sample.

## Data Availability

The datasets analysed in this study are available from the corresponding author on reasonable request.
